# Clinical Impact of Self-Recognition of Recurrent Acute Myocardial Infarction: From KRMI-RCC

**DOI:** 10.3390/jcm13164840

**Published:** 2024-08-16

**Authors:** Kyehwan Kim, Moojun Kim, Chang-Ok Seo, Hangyul Kim, Hye Ree Kim, Min Gyu Kang, Jin-Sin Koh, Jeong Rang Park, Rock Bum Kim, Dong Ryeol Ryu, Jang Hoon Lee, Moo Hyun Kim, Tae-Jin Youn, Dae Woo Hyun, Shin-Jae Kim, Sang Jae Rhee, Sang-Don Park, Young Joon Hong, Jae-Geun Lee, Pil Sang Song, Sang Min Kim, Seung Jin Lee, Jin-Yong Hwang

**Affiliations:** 1 Division of Cardiology, Department of Internal Medicine, Gyeongsang National University School of Medicine, Gyeongsang National University Hospital, Jinju 52727, Republic of Korea; nicol2000@nate.com (K.K.); dxtx73@gmail.com (M.K.); changokseo@gmail.com (C.-O.S.); 13medicine@naver.com (H.K.); hrmanse@naver.com (H.R.K.); med2floyd@naver.com (M.G.K.); kjs0175@gmail.com (J.-S.K.); park-jr@nate.com (J.R.P.); 2 Regional Cardiocerebrovascular Disease Center, Gyeongsang National University Hospital, Jinju 52727, Republic of Korea; krb747@gmail.com; 3 Division of Cardiology, Department of Internal Medicine, Kangwon National University School of Medicine, 156 Baengnyeong-ro, Chuncheon 24289, Republic of Korea; rdr0203@hanmail.net; 4 Department of Internal Medicine, Kyungpook National University Hospital, Kyungpook National University School of Medicine, Kyungpook National University, 807, Hoguk-ro, Buk-gu, Daegu 41404, Republic of Korea; ljhmh75@knu.ac.kr; 5 Department of Cardiology, Dong-A University Hospital, 26, Daesingongwon-ro, Seo-gu, Busan 49201, Republic of Korea; kimmh@dau.ac.kr; 6 Cardiovascular Center, Seoul National University Bundang Hospital, Seoul National University College of Medicine, 173-82, Gumi-ro, Bundang-gu, Seongnam-si 13620, Republic of Korea; ytjmd@snubh.org; 7 Department of Internal Medicine, Andong General Hospital, 11, Angsil-ro, Andong 36743, Republic of Korea; daewoohyun@naver.com; 8 Division of Cardiology, Ulsan University Hospital, University of Ulsan College of Medicine, 171-19, Wolpyeong-ro, Nam-gu, Ulsan 44686, Republic of Korea; kimsc226@uuh.ulsan.kr; 9 Regional Cardiocerebrovascular Center, Department of Cardiovascular Medicine, Wonkwang University Hospital, 33-13 Iksan-daero, Iksan 54536, Republic of Korea; shururuka73@gmail.com; 10 Division of Cardiology, Department of Internal Medicine, Inha University Hospital, 27, Inhang-ro, Jung-gu, Incheon 22332, Republic of Korea; denki1@hanmail.net; 11 Division of Cardiology, Chonnam National University Hospital, 42, Jebong-ro, Dong-gu, Gwangju 61469, Republic of Korea; hyj200@hanmail.net; 12 Division of Cardiology, Department of Internal Medicine, Jeju National University School of Medicine, Jeju National University Hospital, 13-15, Aran, Jeju 63241, Republic of Korea; tedljg@naver.com; 13 Department of Cardiology, Chungnam National University Hospital, 99, Daehak-ro, Yuseong-gu, Daejeon 34134, Republic of Korea; pssong@cnuh.co.kr; 14 Regional Cardiovascular Disease Center, Chungbuk National University Hospital, 776, Sunhwan-ro, Seowon-gu, Cheongju 28644, Republic of Korea; masuri75@hanmail.net; 15 Division of Cardiology, Department of Internal Medicine, Soonchunhyang University Cheonan Hospital, 44, Suncheonhyang 4-gil, Dongnam-gu, Cheonan-si 31151, Republic of Korea; drlsj@schmc.ac.kr

**Keywords:** myocardial infarction, recognition, decision making, process assessments (health care)

## Abstract

**Background/Objectives**: Self-recognition of recurrent myocardial infarction (re-MI) may be essential for reducing prehospital time contrast to awareness of re-MI symptoms. However, data on the current status and clinical impact of self-recognition of re-MI are limited in the contemporary period. Thus, this study aimed to increase this body of knowledge. **Methods**: We enrolled 1018 patients with re-MI using data from the Korean Registry of Acute Myocardial Infarction for Regional Cardiocerebrovascular Centres. The patients were classified into self-recognised MI and unrecognised MI groups, and the differences between them were compared. **Results**: The rate of self-recognition among the patients with previous experience of MI was only 52.4%. Among the patients with re-MI, factors associated with self-recognition included recent first MI within 3 years, prior dyslipidaemia, two or more MI symptoms, and the male gender (*p* < 0.05). Factors associated with a lack of recognition were older age (≥70 years), prior stroke, and cancer history (*p* < 0.05). The proportion of symptoms-to-emergency room arrival time within 90 min among the patients with ST-elevation MI was significantly higher in the self-recognised group than in the unrecognised group (52.6% vs. 31.6%, *p* < 0.001). The self-recognised group showed a lower in-hospital mortality rate (1.5% vs. 6.2%, *p* < 0.001), and this benefit was maintained even after 1 year (hazard ratio: 0.53; *p* < 0.001). **Conclusions**: Only half of the patients who previously experienced a MI recognised a re-MI when it occurred. This recognition reduced prehospital delay and led to higher survival rates, which highlights the importance of patient education as well as objective monitoring devices, irrespective of individual recognition ability for immediate response.

## 1. Introduction

Recurrent myocardial infarction (re-MI) is a common adverse cardiovascular event that occurs after an index event of acute myocardial infarction (MI). Although percutaneous coronary intervention (PCI) dramatically decreases the overall incidence of re-MI, its incidence was reported as 2.5–5.8% at 1 year and 27.1% at 10 years after the initial MI event, and a higher rate of mortality was associated with patients with re-MI compared with those without re-MI [1,2,3]. Re-MI can be prevented by complete functional coronary revascularization, effective dual antiplatelet therapy, and secondary prevention strategies [4]. Similar to first MI events, the most crucial treatment for re-MI is prompt revascularization, which reduces myocardial damage and prevents complications, such as heart failure (HF) and arrhythmia.

In developed countries, prehospital delay has a greater impact than hospital delays, such as door-to-device time delay, on reducing total ischaemic time [5]. Prehospital time depends on the patient’s decision time and home-to-hospital time, which includes factors such as the availability of the emergency medical service and hospital. The patient’s decision time is affected by patient-related factors, symptom-related factors, and the MI event setting [6]. Patient-related factors include age, sex, comorbidity, and awareness of MI symptoms [7]. Since the severity of symptoms ranges from severe chest pain to atypical, mild, or even no pain, this affects patients’ decisions. The level of knowledge of MI symptoms was reported as higher in cardiac patients than in the general population [8,9], and the belief that symptoms were of cardiac origin was found to be the strongest factor in reducing decision time during an MI [10,11].

Patients with re-MI are expected to have a higher recognition rate, faster response, and shorter prehospital time because they have previously experienced MI symptoms and know how to respond. Therefore, this study aimed to investigate the rate of self-recognition of MI at the time of the event among patients with re-MI and to determine its impact on clinical outcomes using data from the Korean Registry of Acute Myocardial Infarction for Regional Cardiocerebrovascular Centres (KRMI-RCC), a national cardiovascular registration project.

## 2. Materials and Methods

### 2.1. Data Collection and Study Approval

We analysed data obtained from KRMI-RCC [5]. This registry is a prospective, observational, cohort, and multicentre database. It has been operational since December 2015 and was officially registered in July 2016. KRMI-RCC is a government-funded research endeavour aimed at comprehensively investigating multifaceted elements encompassing the prehospital, in-hospital, and post-discharge phases of MI within the contemporary landscape. Its purview extends to encompass patient-centric factors, including socioeconomic status, cardiovascular risk factors, and factors pertinent to patient care and treatment. KRMI-RCC also offers patient education and cardiac rehabilitation programmes to prevent future recurrence and improve patients’ access to treatment, thereby ensuring a high quality of care.

This study was approved by the Research Ethics Review Committee of Gyeongsang National University Hospital (approval number 2016-04-013). The Central Committee Group performed a preliminary review process to confirm no conflicts with other KRMI Cardiovascular Centres’ research and approved access to raw data. Patients provided informed consent before enrolment in our prospective cohort. Trained research nurses conducted all surveys and variable inputs. The Central Support Group at Seoul National University Bundang Hospital handled data management and processing.

We analysed data from 2016 to 2019. As COVID-19 began to spread in 2020, data collection was insufficient. Symptoms of COVID-19 and those related to heart and respiratory diseases, including side effects from subsequent vaccines, were often conflated. This may have affected the recognition of heart disease. Therefore, data from 2020 were excluded from this study. Physicians and nurses collected information on demographics, socioeconomic status (e.g., marital status, education and residency with family), smoking status, and self-reported health status in face-to-face interviews during the index event. Patient registration was recommended to be completed within 24 h whenever possible, to collect pre-admission information through interviews. This included estimating the time and place related to the patient’s symptoms and determining whether the symptoms were self-recognised as a heart attack. Comorbidities, laboratory tests, coronary angiography including intervention, the onset and recognition of MI-related symptoms, and echocardiographic variables (left ventricular ejection fraction, LVEF) were extracted from the KRMI dataset. Follow-up data were reviewed for survival and clinical events in the first year. If an enrolled patient did not visit the hospital registered for the study or the patient’s condition made it difficult to visit the hospital, their condition and clinical events were followed up telephonically.

First MI patients were defined as subjects who experienced MI for the first time. Re-MI was defined as a new MI occurring ≥28 days following discharge for the first or a recent MI. Subjects who did not respond or could not respond to a survey on whether they could recognise an MI event were excluded from the analysis. Cases with cardiac arrest that occurred before admission were also excluded from the analysis because of its potentially detrimental effect on cognitive function, which might lead to inaccurate questionnaire answers.

The raw data obtained from the Central Support Group included seven MI-related symptoms: chest pain, difficulty breathing, cold sweats, radiating pain, vertigo/light-headedness, unconsciousness, and stomach-ache. Self-recognised MI and the classification of MI-related symptoms were entered as closed-ended questions (yes, no, or not available). The definition of self-recognition was based on whether the patient personally attributed their symptoms to cardiac causes. If the patient was uncertain about having a heart attack or believed the symptoms were related to another organ, they were included in the unrecognised group. Self-recognition is not related to knowledge about myocardial infarction symptoms or whether the symptoms exactly match those of a myocardial infarction. Self-recognition, in this context, refers to the patient’s recognition of a myocardial infarction and is not related to their overall knowledge level about myocardial infarction, nor to the types of MI-related symptoms.

### 2.2. Study Population

**Figure 1:**
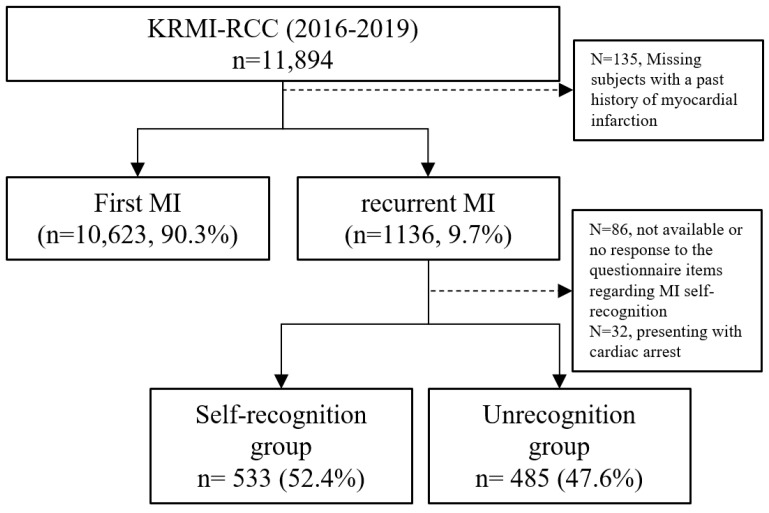
Flow diagram of patient selection for our study using data from the Korean Registry of Regional Cardiocerebrovascular Centres for Acute Myocardial Infarction (KRMI-RCC). MI—myocardial infarction.

We enrolled 11,894 patients registered in KRMI-RCC between 2016 and 2019. Subjects with no recorded history of MI were excluded (n = 135). Among the remaining 11,759 patients, 10,623 (90.3%) experienced their first MI, and 1136 (9.7%) had re-MI. Among the re-MI subjects, 86 patients with missing or null values for a variable related to MI symptoms or self-recognition of MI and 32 cases where cardiac arrest was the presenting symptom were excluded, resulting in a final study cohort of 1018 patients (self-recognised MI group: 533, 52.4%; unrecognised MI group: 485, 47.6%) (Figure 1).

In-hospital events included cardiac arrest during hospitalisation, cerebrovascular accident (CVA; ischaemic or haemorrhagic stroke), cardiogenic shock (systolic blood pressure < 90 mmHg and the use of vasoactive medication), new-onset atrial fibrillation after hospitalisation, HF, ventricular tachycardia, ventricular fibrillation, haemodialysis, and in-hospital death. The definition of new-onset AF included only subjects with newly developed AF after index hospitalisation for myocardial infarction and excluded patients who were aware of atrial fibrillation or were taking medications for atrial fibrillation prior to their hospitalisation. Among the in-hospital cardiovascular clinical events, we were counting only those that occurred after enrolment in our study.

A composite of endpoints was defined as all-cause mortality, cardiovascular death, non-cardiovascular death, non-fatal MI, CVA, and worsening HF. Worsening HF was defined as an increased dose of diuretics or a new initiation of diuretic therapy with evidence of pulmonary and systemic oedema for index hospitalisation. Cardiovascular death was defined as death related to cardiovascular diseases. If the cause of patient death was unclear, it was classified as undetermined. A stroke is defined as a focal or global neurological impairment of sudden onset, lasting more than 24 h (or leading to death), and of presumed vascular origin. Clinical outcomes were assessed by receiving an accurate diagnosis related to composite endpoints through a regional cardiovascular centre registered in this study during the follow-up period or by investigating cardiovascular events through a telephone connection.

### 2.3. Statistical Analysis

The study cohort was divided into a self-recognised MI group and an unrecognised MI group. Categorical variables were expressed as numbers with percentages, and continuous variables were expressed as mean ± standard deviation or median and interquartile range. To compare the self-recognition rate in the first MI group with re-MI groups, we excluded patients presenting with cardiac arrest at the time of admission and those with unavailable or no response variables in the survey, resulting in a sample size of 9666 patients. These data were finally compared with the re-MI patient group (n = 1018) that was analysed in this current study. The same exclusion criteria were applied to both groups.

To identify independent factors affecting MI self-recognition, multivariate analysis was performed for variables with a *p*-value < 0.05, which included the following: male, age < 70 years, college/university graduate, living with a spouse, self-reported dyslipidaemia, stroke, prior KRMI-RCC registration, a history of cancer, prior PCI, and ≥2 MI-related symptoms.

A Kaplan–Meier curve was used to compare the survival rate and a composite of endpoints at 1-year according to the self-recognised or unrecognised group, and the statistical values were calculated using the log-rank test. Additionally, to confirm the independence of all-cause mortality and a composite of endpoints at 1 year, we computed it using the Cox proportional hazards model. The included variables in the Cox proportional hazards model were male age, education level, living with a spouse, dyslipidaemia, prior stroke, prior KRMI-RCC registration, a history of cancer, prior PCI, and two more MI symptoms. The survey regarding cardiovascular outcomes was conducted for all patients one year after the diagnosis of myocardial infarction, but in some cases, the survey could not be conducted due to a lack of contact. Among all enrolled patients, a total of 786 subjects were completely investigated for survival, death, developing a cardiovascular disease, or other medical status at 1 year. *p*-values < 0.05 were considered statistically significant. All analyses were performed using SPSS v21.0 (IBM Corp., Armonk, NY, USA) and R v4.3.2 software [12].

## 3. Results

### 3.1. Baseline Characteristics of the Self-Recognised MI Group and the Unrecognised MI Group

The rate of self-recognised MI is higher among patients with re-MI than first MI (52.3% vs. 14.4%, *p* < 0.001). Table 1 presents the differences in baseline characteristics between the self-recognised and unrecognised MI groups. The patients in the self-recognised MI group had a significantly higher likelihood of being male, younger, and having elevated blood pressure and were less likely to have worsened HF upon hospital admission than the patients in the unrecognised MI group. There were no significant differences in the final diagnosis of either ST-segment elevation myocardial infarction (STEMI) or non-ST-segment elevation myocardial infarction (NSTEMI) between the two groups (Table 1 and Figure S1).

Regarding cardiovascular risk factors, individuals with a history of dyslipidaemia and those who had previously undergone PCI showed a significantly higher rate of self-recognised MI. In contrast, subjects with a history of stroke or cancer showed a significantly lower rate of self-recognised MI. Moreover, a higher level of education was significantly associated with a greater likelihood of MI recognition. Patients living with family members had higher rates of self-recognised MI than those living alone, with the highest rates observed among those living with their spouses. Notably, patients who had been previously registered in KRMI and experienced re-MI within the 3-year study period (2016–2019) exhibited a significantly higher rate of self-recognised MI (64.7% vs. 52.6%, *p* < 0.001) (Table 1 and Figure S1).

At the time of emergency room (ER) arrival, cardiogenic shock was observed more frequently in the unrecognised MI group than in the self-recognised MI group, although this difference was not statistically significant. However, the rate of acute decompensated HF upon ER admission was significantly higher in the unrecognised group than in the self-recognised group (11.8% vs. 16.5%, *p* = 0.032) (Table 1).

### 3.2. Presenting Symptoms, Patient Response and Revascularization Treatment During re-MI

Chest pain was the most common symptom among all patients with re-MI. Typical symptoms such as chest pain, cold sweats, and radiating pain were more common in the self-recognised MI group compared with the unrecognised MI group (Table 1). Atypical MI-related symptoms, such as vertigo, light-headedness, unconsciousness, and stomach-ache, were more common in the unrecognised MI group. The self-recognised group also had a significantly higher proportion of ≥2 symptoms than the unrecognised MI group.

Among the patients with STEMI (for whom reperfusion time is more critical for mortality reduction), the self-recognised MI group had a higher rate of earlier-response patients (symptom-to-arrival time ≤ 60, ≤90, and ≤120 min) than the unrecognised MI group. Among the patients with NSTEMI, the self-recognised MI group showed a significantly higher rate of symptom-to-ER arrival time ≤90 min (30.2% vs. 21.8%, *p* = 0.015). Notably, the self-recognised MI group was more frequently transferred from other hospitals incapable of performing PCI (Table 1).

Primary PCI was performed in 98.4% of all STEMI patients. The median symptom-to-device time was 47 min shorter in the self-recognised MI group than in the unrecognised MI group (135 vs. 182 min, *p* = 0.018). The time to PCI from ER arrival in patients with NSTEMI was similar between the two groups. The two groups had a similar proportion of left main disease and left anterior descending artery stenosis >75% (Table 1). Additionally, there were no significant differences in the other angiographic findings and treatment patterns.

### 3.3. Multivariate Analysis of Self-Recognised MIs for Clinical Outcomes

Multivariate analysis was conducted by incorporating variables related to demographics, socioeconomic status, educational level, chronic disease, cardiovascular risk factors, number of MI symptoms, and history of coronary intervention to verify the potential impact of self-recognised MI (Table 2).

The results revealed that being male, having a college education or higher, having a history of dyslipidaemia, being previously enrolled in the KRMI registry within 3 years, and presenting with ≥2 MI-related symptoms were independent predictors of the self-recognition of MI. Conversely, an older age (>70 years) and a history of previous stroke or cancer emerged as factors that negatively influenced the self-recognition of MI symptoms. 

### 3.4. Clinical Outcomes between the Self-Recognised MI and Unrecognised MI Groups

Table 3 shows the in-hospital events and 1-year clinical outcomes between the self-recognised MI and unrecognised MI groups. The rate of cardiac arrest, cardiogenic shock, new-onset atrial fibrillation, and de novo HF was significantly higher in the unrecognised MI group than in the self-recognised MI group. Notably, the incidence of cardiac arrest in the unrecognised MI group was 3.1 times higher than in the self-recognised MI group, resulting in an in-hospital mortality rate that was 4.1 times higher in the unrecognised MI group (1.5% vs. 6.2%, *p* < 0.001). The all-cause mortality rate in the unrecognised MI group was twice as high as that in the self-recognised MI group at 1 year (13.5% vs. 6.6%, *p* = 0.001). In detail, the occurrence of non-fatal MI, CVA, and hospitalisation for HF was similar between both groups. In contrast, stent thrombosis was significantly higher in the self-recognised MI group compared with the unrecognised MI group (1.9% vs. 0.0%, *p* = 0.010). The 1-year analysis of the composite of endpoints revealed a significantly higher incidence in the unrecognised MI group compared with the self-recognised MI group (14.6% vs. 21.6%, *p* = 0.004).

**Figure 2:**
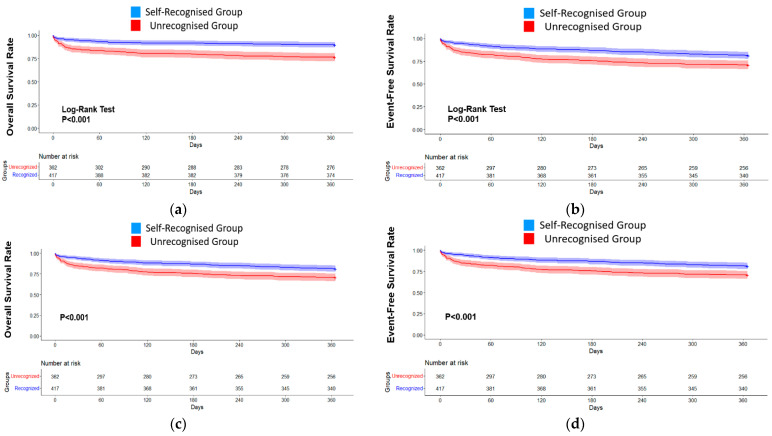
Clinical Outcomes at 1-year follow-up between self-recognition of re-MI and unrecognised patients. The Kaplan–Meier curves demonstrate that the self-recognised group (blue) showed better outcomes ((**a**), all-cause mortality; (**b**), a composite of endpoints) at 1 year than the unrecognised group (red). The Cox proportional hazard models (**c**,**d**) were adjusted according to sex, age, education level, a history of dyslipidaemia, previous KRMI-RCC registration, and two more MI-related symptoms (**c**): all-cause mortality at 1 year (HR = 0.53, 95% CI 0.27–0.65, *p* < 0.001). (**d**) A composite of clinical outcomes at 1 year (HR: 0.77, 95% CI 0.50–0.98, *p* < 0.001).

Cox proportional hazard models revealed that the self-recognised MI group independently exhibited better 1-year outcomes for all-cause mortality (HR 0.53, 95% CI 0.27–0.65, *p* < 0.001, Figure 2c) and the composite of endpoints (HR 0.77, 95% CI 0.50–0.98, *p* < 0.001, Figure 2d) compared with the unrecognised MI group.

## 4. Discussion

There are numerous studies on awareness of MI symptoms in the general population and patients with ischemic heart disease except re-MI. However, few data are available on MI recognition in re-MI patients. To the best of our knowledge, this is the first report on the self-recognition for MI at the time of re-MI and its clinical impact on prognosis. The summary of this study is as follows: (1) The rate of self-recognised MI was only 14.4% among patients experiencing their first MI and 52.4% among patients with re-MI. (2) Among the patients with re-MI, the factors associated with self-recognition of MI were prior KRMI hospital admission (re-MI within 3 years), prior dyslipidaemia, the male gender, and a high burden of MI symptoms. Factors negatively influencing self-recognition were older age, prior stroke, and cancer history. (3) The in-hospital mortality rate in the unrecognised MI group was 4.1 times higher than that in the self-recognised MI group, and the number of undesirable events, including cardiac arrest, cardiogenic shock, and new-onset atrial fibrillation, was also higher. All-cause mortality at 1 year was also significantly higher in the unrecognised MI group than in the self-recognised MI group. It is unknown whether patients who experienced re-MI with severe complications believed that it was not myocardial infarction because they experienced different symptoms from those they experienced during their first MI or whether they had an impaired ability to recognise myocardial infarction.

Despite many interventional research studies and trials, prehospital delay was reported as unchanged (median, 2–4 h) over a 20-year observation period [13]. The prehospital delay includes patient decision time delay (time from symptom onset to calling for medical assistance) and home-to-hospital delay (time from calling for help to hospital admission), and the patient’s decision time delay accounts for almost two-thirds of prehospital delay [11]. The rate of self-recognised heart attack symptoms was reported as 26% among 228 patients with acute coronary syndrome [11] and 67% among 521 patients with STEMI [6]. Surprisingly, although awareness of MI symptoms in a disease-free period may impact self-recognition of MI [14], the rate of self-recognised MI in our study was notably low (14.4%) among patients experiencing their first MI and was much lower than the rate of awareness of MI symptoms in the Korea Community Health Survey, which reported that 69.9% of participants who were aware of typical symptoms (chest pain) stated that they would call 911 [10]. Compared with patients experiencing their first MI, the rate of self-recognised MI was considerably higher among patients with re-MI because after experiencing their first MI, patients might gain further understanding of MI, leading to a higher rate of self-recognition. Unfortunately, the half of the re-MI patients did not recognise MI at the time, which might contribute to a delay in decision time and prehospital time. Current educational efforts for secondary prevention after first MI may have the room to improve patients’ behaviour during a re-MI. In our study, the self-recognition of MI at the time of the event may reduce prehospital delay, resulting in better clinical outcomes. Thus, all educational efforts to reduce prehospital delay need to focus on the self-recognition of re-MI at the time of the event beyond awareness of MI symptoms.

The self-recognition of MI is intricately intertwined with patient-related factors, symptom-related factors, and the setting of the event. A longer decision time and prehospital time have been associated with the female sex, older age, living alone, minority ethnic groups, a low education level, history of chronic disease, and lack of knowledge of symptoms [8,11]. A report from Swedish hospitals on factors associated with patient decision time in STEMI showed that the belief that symptoms were of cardiac origin had the highest association with help from early responders arriving in less than 20 min (OR: 2.6). Furthermore, associated symptoms, such as dyspnoea (OR: 1.67) and weakness (OR: 1.65), were associated with early response, whereas chest pain was not independently associated with response time. Cold sweats (OR: 0.61) prevented late care-seeking behaviour, as did a high symptom burden (OR: 0.86) [6]. The occurrence of an MI at home or outside office hours may delay the decision time, and the presence of a bystander may shorten the decision time [6,11]. The findings in our study showed that typical symptoms such as chest pain, cold sweats, and radiating pain were more common and atypical MI-related symptoms such as vertigo, light-headedness, unconsciousness, and stomach-ache occurred less in the self-recognised MI group compared with the unrecognised MI group. This suggests that previously experienced typical symptoms may be more likely to be attributed to a heart attack, while atypical symptoms were related to decreased MI recognition rates like the results from the first MI. Notably, patients who have previously enrolled in the KRMI registry should receive an educational programme on MI by trained nurses, and those who had a re-MI within the 3 years of our study had a higher rate of self-recognised MI. Among the 481 patients enrolled in this study, patients who had previously been enrolled had a high rate of MI recognition. In particular, typical symptoms (chest pain; [88.5% vs. 89.7%, *p* = 0.552]; cold sweats; [27.9% vs. 23.9%, *p* = 0.154]; radiating pain; [23.4% vs. 24.4%, *p* = 0.715]) have been registered. There was no statistical difference compared to the patient group, but atypical symptoms (respiratory distress; 36.1% vs. 29.4%, *p* = 0.026; stomach-aches; 2.3% vs. 0.7%, *p* = 0.047) were significantly more frequent. It might be assumed that previously registered KRMI patients acquired greater MI-related knowledge, which, in turn, was linked to a relatively short prehospital delay, which may have influenced clinical outcomes compared with the unrecognised group [15]. Additionally, even among patients who had previously experienced myocardial infarction, one-third of those in the unrecognised group did not exhibit typical symptoms, with an average of only 1.8 symptom types reported. This implies that self-recognition of MI patients may often need to be made based on just one or two symptoms. Moreover, even if chest pain, a well-known typical symptom, is present, its mildness, difference from previous experiences, or ambiguity may make it challenging to recognise as a myocardial infarction. To improve the rate of recognition of re-MI from the result of this study, we suggest that patient education should focus on the recognition of re-MI attack, including knowledge expansion from typical symptoms to atypical symptoms and more intensive and repetitive education among the vulnerable patients for self-recognition. Education programmes for MI patients are expected to be cost-effective because the incidence of re-MI and mortality are higher in those who have experienced their first MI than in the general population. Further studies must focus on a well-designed educational programme to enhance self-recognition of MI and its impact on clinical outcomes among patients with re-MI. On the other hand, even if a patient has already experienced a myocardial infarction, the patients who recognised the myocardial infarction only identified it because of atypical symptoms and patient factors. Therefore, as MI treatment develops daily, interest in re-MI patients increases, and public awareness and education require much attention. This study demonstrates that relying solely on patient perception may have limitations in terms of managing myocardial infarction. It highlights the need for objective monitoring tools to recognise myocardial infarction. Campaigns to raise awareness increased first medical contact/EMS use, which was linked to mortality rates [16]. There are certainly limits to relying on the patient’s experience and knowledge to make decisions. Therefore, improving awareness/knowledge of myocardial infarction and providing continuous education and feedback to patients with recurrent myocardial infarction seems necessary. Additionally, AI and wearable devices that have been actively researched recently may be an alternative that can overcome the limitations of unmet needs through ECG monitoring or early detection of MI [17].

Our study had several limitations. First, the investigation relied on patients’ memories, possibly introducing recall bias. Because we relied on the patient’s memory, only communicable patients were enrolled during the enrolment process, while some patients with cognitive impairment and patients who visited the hospital while experiencing cardiac arrest were excluded, although their cognitive function may have been later confirmed to be normal. Therefore, there may be missing subjects, which may have resulted in selection bias. Additionally, we were unable to discern whether education due to a prior MI had influenced patients with re-MI or if their recognition of MI symptoms was solely based on personal experience. Furthermore, precise details of MI-related symptoms, such as the location, intensity, and duration, were not obtained. The analysis solely focused on whether patients recognised their symptoms as MI-related without investigating if they had attributed them to other causes. This registry study could have accurately assessed the level of knowledge about myocardial infarction symptoms through a survey on patients’ awareness of myocardial infarction. The correlation between each symptom and the attribution of MI was not ascertainable from the available data. Specific details regarding the prehospital delay, including the time from symptom recognition to medical contact or hospital admission, could not be precisely determined. Moreover, relying on patients’ recollections to record the time of symptom onset may have introduced inaccuracies in the recorded time intervals. Another limitation is the lack of information about previous MI affecting recognition of re-MI, such as the time interval between first MI and re-MI, symptoms type, severity, type of treatment, participation in post-MI education, and compliance with drug treatment (these details were not included in the registry), which will be a new research target for recognition of re-MI. Finally, it is important to note that the survey primarily consisted of closed-ended questions with binary ‘yes’ or ‘no’ responses, which may have posed challenges when it came to accurately assessing the depth of understanding of specific symptoms.

## 5. Conclusions

The data analysis from a large-scale national registry revealed that only 14% of patients experiencing their first MI and 52.4% of re-MI patients recognised the symptoms at the time of the event as related to a heart attack. Patients with re-MI who self-recognised symptoms had a shortened prehospital time and such recognition was associated with reduced rates of in-hospital and 1-year mortality. Therefore, an educational programme providing a high level of MI knowledge, including the typical and atypical symptoms of MI, must be undertaken for secondary prevention, and the lack of ability to recognise a MI even in the patients who have previously experienced MI may highlight objective monitoring and immediate response systems in the future.

## Figures and Tables

**Table 1:** Demographic and clinical characteristics between the self-recognised MI group and the unrecognised MI group.

	Self-Recognised N = 533 (52.4%)	Unrecognised N = 485 (47.6%)	*p*-Value
Female	112 (20.7%)	158 (31.0%)	<0.001
Age	67 [59, 75]	73 [64, 80]	<0.001
Body mass index, kg/m^2^	23.9 [22.1, 26.0]	23.7 [21.5, 26.1]	0.380
Diagnosis			0.677
STEMI	140 (26.3%)	133 (27.4%)	
NSTEMI	393 (73.7%)	352 (72.6%)	
Systolic blood pressure, at arrival	130 [110, 150]	128 [110, 150]	0.003
Diastolic blood pressure, at arrival	80 [70, 90]	75 [60, 90]	<0.001
Heart rate, at arrival (beats per minute)	78 [67, 92]	80 [67, 94]	0.321
Cardiogenic shock at arrival	19 (3.6%)	28 (5.8%)	0.094
Worsened heart failure at arrival	63 (11.8%)	80 (16.5%)	0.032
Cardiovascular risk factors			
Hypertension	341 (64.0%)	309 (63.7%)	0.930
Dyslipidaemia (patient-reported)	107 (20.1%)	66 (13.6%)	0.006
Diabetes mellitus	251 (47.1%)	225 (46.4%)	0.823
On dialysis	40 (7.5%)	50 (10.3%)	0.115
Prior PCI	494 (92.7%)	431 (88.9%)	0.035
Prior CABG	31 (5.8%)	22 (4.5%)	0.359
Prior stroke	43 (8.1%)	75 (15.5%)	<0.001
Prior PAD	7 (1.3%)	4 (0.8%)	0.552
Previously registered subjects	345 (64.7%)	255 (52.6%)	<0.001
Prior cancer	21 (3.9%)	44 (9.1%)	0.001
Smoking			0.211
Current smoker	147 (27.6%)	116 (23.9%)	
Ex-smoker	157 (29.5%)	135 (27.8%)	
Never	229 (43.0%)	234 (48.2%)	
Type of Insurance			0.661
Medical insurance	493 (92.5%)	441 (90.9%)	
Medical care or non-benefits	40 (7.5%)	44 (9.1%)	
Education levels			0.001
Elementary school	120 (22.5%)	148 (30.5%)	
Middle school	65 (12.2%)	80 (16.5%)	
High school	184 (34.5%)	149 (30.7%)	
College/university	116 (23.9%)	65 (14.7%)	
Unknown	48 (9.0%)	43 (8.9%)	
Types of Households			0.067
Solitary or alone	90 (16.9%)	110 (22.7%)	0.020
Living with family	433 (81.2%)	366 (75.5%)	0.025
with spouse	376 (70.5%)	296 (61.0%)	0.001
with parents	122 (22.9%)	115 (23.7%)	0.757
with son or daughter	111 (20.8%)	112 (23.1%)	0.382
with housemate	10 (1.9%)	9 (1.9%)	0.981
Laboratory findings			
eGFR, mL/min/1.73 m^2^	72 [53, 89]	69 [42, 91]	0.003
Haemoglobin, g/dL	13.5 [11.7, 14.9]	12.7 [10.9, 14.2]	<0.001
HbA1c, %	6.2 [5.7, 7.3]	6.0 [5.6, 7.0]	0.033
Total cholesterol, mg/dL	137 [114, 162]	137 [113, 174]	0.281
High-density lipoproteinemia, mg/dL	40 [34, 48]	40 [34, 48]	0.281
Low-density lipoproteinemia, mg/dL	78, [61, 98]	80 [61, 108]	0.235
LVEF, %	50 [41, 59]	48 [37, 57]	0.003
LVEDD, mm	51 [47, 55]	51 [46, 56]	0.640
Recognised symptoms at the development of acute myocardial infarction			
Chest pain	495 (92.9%)	410 (84.7%)	<0.001
Difficult breathing	171 (32.1%)	158 (34.7%)	0.386
Cold sweats	167 (31.4%)	100 (20.7%)	<0.001
Radiating pain	146 (27.4%)	96 (19.8%)	0.004
Vertigo/light-headedness/unconsciousness	45 (8.6%)	80 (16.3%)	0.004
stomach-ache	7 (1.3%)	10 (2.1%)	0.352
Number of MI symptoms	1.9 ± 0.9	1.8 ± 0.9	0.008
(2 or more)	342 (64.2%)	270 (55.7%)	0.006
Place where symptoms occurred			0.915
At home	361 (67.7%)	330 (68.0%)	
Outside of the home	172 (32.3%)	155 (32.0%)	
Symptom to arrival time, median, min	139 [60, 338]	190 [91, 661]	0.056
≤60 min	122 (25.2%)	87 (19.8%)	0.051
≤90 min	177 (36.5%)	109 (24.8%)	<0.001
≤120 min	223 (46.0%)	154 (35.0%)	<0.001
Among STEMI, median, min	84 [40, 182]	127 [61, 291]	0.017
≤60 min	56 (40.9%)	33 (24.8%)	0.005
≤90 min	72 (52.6%)	42 (31.6%)	<0.001
≤120 min	92 (67.2%)	62 (46.6%)	0.001
Among NSTEMI, median, min	180 [74, 414]	230 [104, 852]	0.242
≤90 min	105 (30.2%)	67 (21.8%)	0.015
Methods of transportation to the PCI-capable hospital			0.204
Ambulance	298 (56.1%)	303 (62.5%)	
Own car	188 (35.4%)	154 (31.8%)	
Public transport	35 (6.6%)	24 (5.0%)	
On foot	9 (1.7%)	4 (0.8%)	
Inter-hospital transfer	183 (36.0%)	122 (22.6%)	<0.001
Performed PCI	467 (87.6%)	416 (85.8%)	0.386
Use for a mechanical support device	11 (2.1%)	12 (2.5%)	0.660
Treatment status of STEMI			0.355
Primary PCI	120 (98.4%)	112 (97.4%)	
Thrombolysis	1 (0.8%)	0 (0.0%)	
Conservative Tx	1 (0.8%)	3 (2.6%)	
Symptoms to Device time, min (median)	135 [98, 225]	182 [117, 313]	
Symptoms to Device time, min (avg.)	196 ± 192	591 ± 1730	0.018
Treatment status of NSTEMI *			
PCI within 24 h from arrival *	244 (62.1%)	197 (56.0%)	0.173
Conservative Therapy	43 (10.9%)	54 (15.3%)	
Angiographic findings			
Infarct-related artery			0.844
Left anterior descending artery	197 (37.0%)	178 (36.7%)	
Right coronary artery	145 (27.2%)	139 (28.7%)	
Left circumflex artery	94 (17.6%)	75 (15.5%)	
Left main	18 (3.4%)	15 (3.1%)	
Unknown or undetermined	79 (14.8%)	78 (16.1%)	
The number of vessels			0.054
0	24 (5.1%)	12 (2.9%)	
1	259 (55.5%)	206 (49.5%)	
2	127 (27.2%)	137 (32.9%)	
3	57 (12.2%)	61 (14.7%)	
Left main disease	44 (9.4%)	34 (8.1%)	0.757
Left anterior descending artery stenosis ≥ 75%	151 (32.3%)	155 (37.2%)	0.256
Complete revascularization	375 (80.3%)	324 (77.9%)	0.378
Performed bypass surgery	8 (1.5%)	8 (1.6%)	0.849

STEMI—ST-segment elevation myocardial infarction; NSTEMI—non-ST-segment elevation myocardial infarction; PCI—percutaneous coronary intervention; CABG—coronary artery bypass graft; PAD—peripheral artery disease; eGFR—estimated glomerular filtration rate (calculated using the modification of diet in renal disease equation); HbA1c—glycated haemoglobin; LVEF—left ventricular ejection fraction; LVEDD—left ventricular end-diastolic dimension; MI—myocardial infarction. * This was calculated from 538 (72.2%) NSTEMI patients undergoing PCI because of missing data.

**Table 2:** Binary logistic analysis for factors associated with the self-recognition of re-MI.

Variables	Multivariate Analysis		
	OR	95% CI	*p*-Value
Male	1.55	1.10–2.19	0.013
Age ≥ 70-year-old	0.57	0.42–0.76	<0.001
Education beyond university	1.41	0.98–2.03	0.062
Living with spouse	1.28	0.94–1.74	0.116
Prior dyslipidaemia	1.58	1.08–2.30	0.018
Prior stroke	0.54	0.35–0.84	0.006
Previously enrolled KRMI-RCC	1.78	1.35–2.36	<0.001
A history of cancer	0.33	0.18–0.61	<0.001
Prior PCI	1.34	0.82–2.19	0.243
MI symptoms (2 or more)	1.39	1.05–1.84	0.021

OR—odds ratio; CI—confidence interval; KRMI-RCC—Korean Registry of Acute Myocardial Infarction for Regional Cardiocerebrovascular Centres; PCI—percutaneous coronary intervention; MI—myocardial infarction. Previously enrolled KRMI-RCC refers to subjects who previously visited the relevant regional cardiocerebrovascular institution.

**Table 3:** Differences between the self-recognised MI group and unrecognised MI group for in-hospital and 1-year clinical outcomes after discharge.

	Self-Recognition N = 533	Unrecognition N = 485	*p*-Value
Hospital stay, days	8 ± 9	7 ± 18	0.235
In-hospital events			
Cardiac arrest	10 (1.9%)	28 (5.8%)	<0.001
Cerebrovascular event	1 (0.2%)	4 (0.8%)	0.159 *
Cardiogenic shock	23 (4.3%)	43 (8.9%)	0.003
Atrial fibrillation	33 (6.2%)	51 (10.5%)	0.012
Heart failure	57 (10.7%)	71 (14.6%)	0.036
Ventricular tachycardia or ventricular fibrillation	23 (4.3%)	29 (6.0%)	0.144
Need for haemodialysis	34 (6.4%)	37 (7.6%)	0.255
In-hospital death	8 (1.5%)	30 (6.2%)	<0.001
A composite of in-hospital events (cardiac arrest, cardiogenic shock, stroke, heart failure, ventricular fibrillation/tachycardia, haemodialysis, and cardiac death)	111 (20.8%)	144 (29.7%)	0.001
Clinical outcomes at 1-year follow-up after discharge *	N = 417	N = 362	
All-cause mortality	28 (6.6%)	48 (13.5%)	0.001
Cause of death			0.029
Cardiac death	15 (53.6%)	25 (52.1%)	
Non-cardiac death	12 (42.9%)	11 (22.9%)	
Undetermined	1 (3.6%)	12 (25.0%)	
Non-fatal myocardial infarction	34 (7.9%)	17 (4.8%)	0.051
Cerebrovascular accident	4 (0.9%)	5 (1.4%)	0.529
Rehospitalisation for heart failure	14 (3.2%)	12 (3.4%)	0.913
Stent thrombosis	8 (1.9%)	0 (0.0%)	0.010
A composite of all clinical events at 12-month follow-up (all-cause death, stroke, heart failure, myocardial infarction)	78 (14.6%)	105 (21.6%)	0.004

* Outcomes of 786 patients at 1 year.

## Data Availability

After deliberation, the data presented in this study are available upon request from the KRMI-RCC Steering Committee. Data availability is restricted to participating researchers and, therefore, unavailable to the public.

## Supplementary Materials

The following supporting information can be downloaded at: https://www.mdpi.com/article/10.3390/jcm13164840/s1, Figure S1: Clinical outcomes and features between the self-recognised MI group and unrecognised MI group.
