# Supplementary material for: Clinical Impact of Self-Recognition of Recurrent Acute Myocardial Infarction: From KRMI-RCC
Source: J Clin Med. 2024 Aug 16;13(16):4840. doi: 10.3390/jcm13164840 (PMC11355230; doi:10.3390/jcm13164840)

Supplementary Figure S1 Clinical outcomes and features between the self-recognized MI group and unrecognized MI group

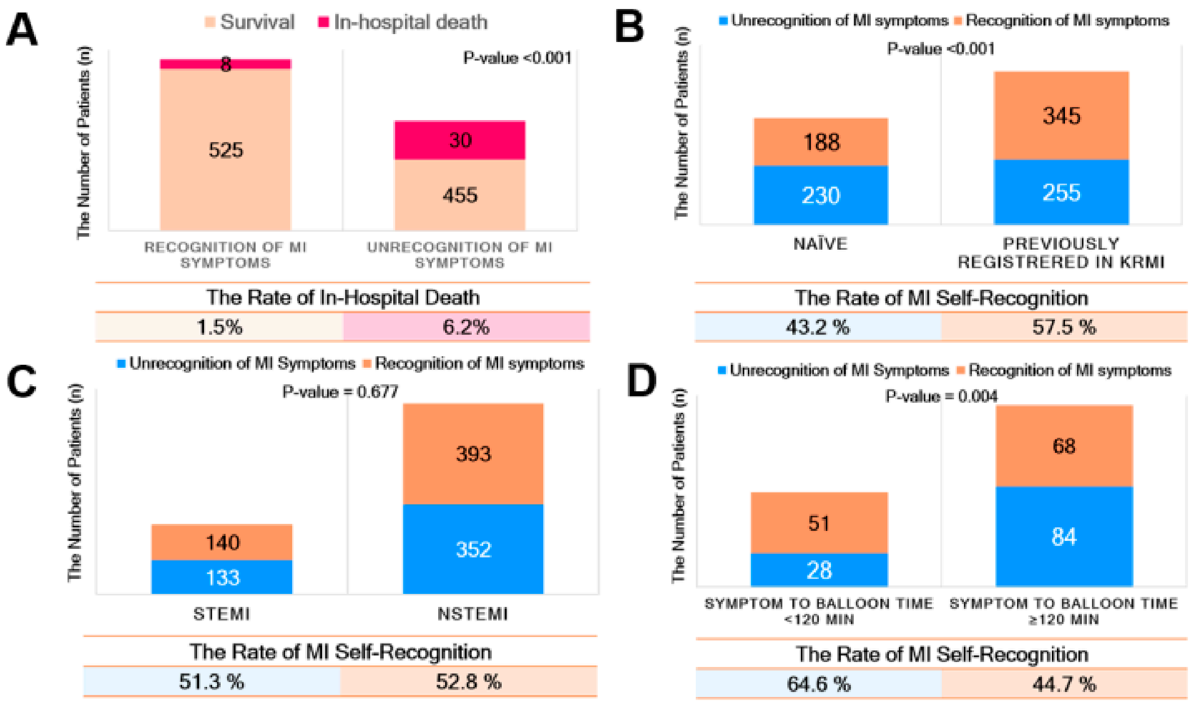

Supplement: Supplementary file 1 [file jcm-13-04840-s001.zip › jcm-3101566-supplementary.pdf]
